# Acceptability of Multiple Micronutrient-Fortified Bouillon Cubes among Women and Their Households in 2 Districts in The Northern Region of Ghana

**DOI:** 10.1016/j.cdnut.2023.102056

**Published:** 2023-12-08

**Authors:** K. Ryan Wessells, Sika M Kumordzie, Emily Becher, Jennie N Davis, Kania W Nyaaba, Sarah J Zyba, Charles D Arnold, Xiuping Tan, Stephen A Vosti, Katherine P Adams, Marjorie Haskell, Seth Adu-Afarwuah, Reina Engle-Stone

**Affiliations:** 1 Institute for Global Nutrition, University of California, Davis, Davis, CA, United States; 2 Department of Nutrition, University of California, Davis, Davis, CA, United States; 3 Department of Nutrition and Food Science, University of Ghana, Legon, Accra, Ghana; 4 School of Public Health, University of California, Berkeley, Berkeley, CA, United States; 5 Department of Agricultural and Resource Economics, University of California, Davis, Davis, CA, United States

**Keywords:** micronutrient, large-scale food fortification, bouillon, deficiency, vitamin, mineral, acceptability, women

## Abstract

**Background:**

Bouillon is a promising large-scale food fortification vehicle, but there is uncertainty regarding the types and concentrations of micronutrients that are feasible to add without compromising consumer acceptability.

**Objective:**

The objective of this study was to evaluate the acceptability of 2 different multiple micronutrient-fortified bouillon cube formulations, compared with a bouillon cube fortified with iodine only.

**Methods:**

We conducted a double-blind, randomized, controlled acceptability study in 2 districts in northern Ghana. Two nonproprietary, noncommercialized formulations of multiple micronutrient-fortified bouillon cubes containing iron, zinc, folic acid, vitamins A and B12, and iodine at “upper-level” (45-125% CODEX NRV/2.5g) or “lower-level” (15-50% CODEX NRV/2.5g) concentrations, and a control cube that contained iodine only (50% CODEX NRV/2.5g) were evaluated. Eligible women (*n* = 84) were invited to participate in *1)* center-based sensory evaluations designed to permit within-individual comparisons among the different study products; and *2)* in-home evaluation of bouillon acceptability and use, in which participants were randomized to receive 1 of the 3 study products to use in household cooking for a 2-wk period. Acceptance test ratings were based on a 5-point Likert scale (1 = dislike very much, 5 = like very much).

**Results:**

In the center-based evaluations, overall liking of the 3 bouillon cube formulations both dry and in prepared dishes ranged from 4.3 to 4.6 on the 5-point Likert scale and did not differ among formulations (*P* > 0.05). After the 2-wk in-home trial, 93.8% of index participants (*n* = 75/80) rated their overall liking of the bouillon product formulation to which they were randomly assigned as “like” or “like very much” (4–5 on the 5-point Likert scale) and median apparent intake of study-provided bouillon over 2 wk was 3.6 g/capita/d; neither value differed by study group (*P* = 0.91 for both).

**Conclusions:**

All 3 formulations of bouillon cubes assessed were acceptable to women and their households in 2 districts in northern Ghana.

This trial was registered at www.clinicaltrials.gov as NCT05177614.

## Introduction

Anemia and micronutrient deficiencies are severe and widespread in sub-Saharan Africa, particularly among young children and women of reproductive age [1]. A recent analysis by Stevens et al. estimated that 62% of preschool children and 80% of women of reproductive age in sub-Saharan Africa have at least one micronutrient deficiency (i.e., iron, zinc, or vitamin A for preschool children; iron, zinc, or folate for women of reproductive age) [1]. There is strong evidence for the implementation of several different interventions to address micronutrient deficiencies, including high-dose vitamin A supplementation in preschool-aged children, multiple micronutrient supplementation during pregnancy, and large-scale fortification of commonly consumed foods and condiments (e.g., salt, sugar, oil, and flour) with micronutrients [2]. However, existing programs may not reach all population groups at risk for deficiency or deliver enough micronutrients to achieve dietary adequacy. Thus, given the extent of deficiency, additional strategies for addressing micronutrient deficiencies may be warranted, including large-scale food fortification of other food vehicles [[3], [4], [5]].

Bouillon is a promising food fortification vehicle in West Africa because the product is centrally processed on a large scale and consumed by the majority of households (including rural households of low socio-economic status) [[6], [7], [8], [9]]. Surveys in 4 West African countries reported that 79 to 99% of women had consumed bouillon in the last week [10], whereas a more recent survey of households in 2 states of Nigeria (Ebonyi and Sokoto) found that ≥ 99% of households reported using bouillon cubes to prepare foods [11]. A recent pilot survey in northern Ghana found that 99% of respondents had ever cooked with bouillon, and 77% reported typically cooking with bouillon ≥ twice/d [12]. Commercial bouillon cubes are generally fortified with iodized salt [8], and some multinational companies currently include additional micronutrients, such as vitamin A (∼48 μg/g bouillon) or iron (∼0.6 mg /g bouillon), on a voluntary basis [8, 13, 14]. Initial results from modeling of national survey data from several countries (e.g., Cameroon, Ghana, and Haiti) suggest that multiple micronutrient fortification of bouillon could improve dietary adequacy, albeit at micronutrient fortificant concentrations typically greater than those currently being used for voluntary fortification [15].

The West Africa Condiment Micronutrient Innovation Trial (CoMIT) project aims to address some of the evidence gaps related to multiple micronutrient fortification of bouillon and includes a randomized controlled trial to evaluate the effects of multiple micronutrient-fortified bouillon cubes on adequacy of intake and biomarkers of micronutrient status among vulnerable individuals (women of reproductive age and children 2–5 y old) within households [16]. This trial will provide evidence that could be used to guide discussions of voluntary or mandatory standards for the multiple micronutrient fortification of bouillon. Development of a multiple micronutrient-fortified bouillon formulation (including concentrations and chemical forms of the fortificants) for evaluation in a clinical trial must consider both the micronutrient requirements of different populations and the technical compatibility of the fortificants with the food matrix while also ensuring consumer acceptability. However, consumer acceptability of bouillon cubes fortified with micronutrients at concentrations greater than those used in voluntary fortification is uncertain.

The present study aimed to address this gap by evaluating the acceptability of 2 different multiple micronutrient-fortified bouillon cubes formulated with 6 micronutrients for which deficiency is common among women and children in Ghana (vitamins A, B9 and B12, iron, zinc, and iodine). The specific objectives were to: *1)* determine the sensory acceptability of the multiple micronutrient-fortified bouillon cubes, compared with control bouillon cubes (fortified with iodine only), before and after preparation in local dishes; *2)* determine the ability of participants to differentiate between the multiple micronutrient-fortified bouillon cubes and control bouillon cubes when presented together; *3)* assess differences in bouillon consumption rates when participants use multiple micronutrient-fortified bouillon cubes compared with control cubes in their own homes, and *4)* assess hypothetical willingness-to-pay (WTP) for multiple micronutrient-fortified bouillon cubes before and after being exposed to them.

## Methods

### Study design

This study evaluated the acceptability of 2 different multiple micronutrient-fortified bouillon cubes, compared with a control cube, through a series of assessments: *(1)* center-based sensory evaluations (affective and difference testing) conducted as a crossover double-blind, randomized placebo-controlled trial designed to permit within-individual comparisons among the different study products; and *(2)* in-home evaluation of the acceptability and use of the study-provided bouillon cubes, conducted as a double-blind randomized controlled trial in which participants were randomly assigned to receive 1 of the 3 study products, and asked to use it in household cooking for 2 wk. To provide additional context, we also conducted focus group discussions to elicit participants’ perspectives and experiences with the study-provided bouillon cubes and assessed hypothetical WTP for multiple micronutrient-fortified bouillon cubes.

The study was conducted in the Kumbungu and Tolon districts in the Northern Region of Ghana from November 2021 to January 2022. Five cohorts of 12 to 18 women per cohort were enrolled over the course of the study.

### Product development

Two nonproprietary, noncommercialized model 10 g shrimp bouillon cubes (MC1C and MC2C), fortified with iodine only, were formulated by an international consortium of bouillon producers and produced by The GB Foods, S.A. (Barcelona, Spain). Shrimp flavoring was selected based on pilot work in northern Ghana, where 87% of household surveys reported consuming commercially available shrimp-flavored bouillon without other spices most frequently [12]. The 2 model cubes differed only in the flavor profile of the shrimp top notes (i.e., aromas and tastes perceived first by the consumer). Consumer acceptability of the model cubes, compared with a reference commercial cube, was assessed in Northern Ghana by The Commonwealth Scientific and Industrial Research Organization (CSIRO; Australia) and Consumer Insights Consult Ltd (Accra, Ghana). Both bouillon cubes performed well and had high consumer acceptance; the MC1C model cube was selected for further fortification work (Nicholas Archer, CSIRO, personal communication).

Characteristics of the multiple micronutrient-fortified bouillon cubes, including micronutrient levels and specific chemical forms of micronutrients tested in this study, are shown in Table 1 [17,18]. Using national survey data from Ghana and other West African countries [10, 15] and data collected in a pilot survey in the Kumbungu and Tolon districts in Ghana [12], we estimated average daily bouillon consumption to be 2 to 2.5 g/d for women of reproductive age (15–49 y) and 1 g/d for children from 2 to 5 y of age. The concentrations of micronutrients included in the “upper-level” fortified bouillon cubes were selected based on modeling these survey data to estimate the contribution of hypothetical fortification of bouillon on adequacy of dietary micronutrient intake [19, 20], review of the scientific literature on micronutrient interventions, and, for iron and vitamin A, theoretical calculations of accrual of nutrient stores [16, 21]. Based on the aforementioned assumed average daily bouillon consumptions, we expect the “upper-level” multiple micronutrient fortified bouillon to provide ∼33 to 100% of the recommended daily allowance (RDA) of the included micronutrients without exceeding the tolerable upper intake level (UL) [22, 23]. The estimated average contribution exceeds 100% of the RDA only for vitamin B12, for which there is no UL due to any known adverse effects [23]. Due to concerns about potential negative effects of the micronutrients, particularly iron, on the organoleptic profile of the “upper-level” fortified bouillon cubes, the “lower-level” formulation included the same micronutrient profile but with a lower concentration of all micronutrients except iodine. Assuming 2.5 g/d bouillon consumption among women of reproductive age, the micronutrient content of the “lower-level” formulation was set at 30% of the Codex Alimentarius nutrient reference values (NRV) for folic acid, vitamin B12, zinc, and vitamin A and 15% of the Codex Alimentarius NRV for iron [24]. Based on the aforementioned assumed average daily bouillon consumptions for women of reproductive age and children 2 to 5 y of age, we expect the “lower-level” multiple micronutrient fortified bouillon to provide ∼13 to 56% of the RDA of the included micronutrients without exceeding the UL [22, 23].

**TABLE 1:** Chemical form of the fortificant and target fortification levels of the multiple micronutrient-fortified bouillon cubes^1^

Fortificant	Target micronutrient concentration per gram bouillon 2		
	“Upper-level”	“Lower-level”	Control
Vitamin A (retinyl palmitate)	200 μg RE	96 μg RE	---
Folic acid	80 μg	28.8 μg	---
Vitamin B12	1.2 μg	0.288 μg	---
Iron (FePP/CA/TSC)^3^	4 mg	1.3 mg	---
Zinc (ZnO)	3 mg	1.68 mg	---
Iodine (KIO_3_)	30 μg	30 μg	30 μg

1 FePP, iron pyrophosphate; CA, citric acid; TSC, trisodium citrate; RE, retinol equivalent.

2 Target micronutrient concentrations do not include overage values to account for micronutrient loss during storage and cooking. Overage values were selected based on industry experience.

3 FePP has low bioavailability relative to other iron salts [17]; enhancers (i.e., citric acid/trisodium citrate) were added to the micronutrient premix included in the bouillon in an attempt to compensate for this reduced bioavailability [18].

The specific fortificants were selected based on technical compatibility with the food matrix and history of use as fortificants. In the case of iron, the combination of ferric pyrophosphate (FePP) with a citric acid/trisodium citrate buffer (CA/TSC) was expected to enhance bioavailability compared to FePP alone [17]. The micronutrient premix was supplied by DSM Nutritional Products (Johannesburg, South Africa), and vitamin A was supplied by BASF (Copenhagen, Denmark). The multiple micronutrient-fortified and control (fortified with iodine only) bouillon cubes tested in this study were manufactured by The GB Foods, S.A. (Barcelona, Spain), using the nonproprietary MC1C shrimp model cube formulation and stored under controlled conditions. The 10 g bouillon cubes were individually wrapped in white foil and labeled with a 3-digit alpha-numeric study code; investigators and study participants were blinded to the type of product. Micronutrient concentrations of the multiple micronutrient-fortified and control bouillon cubes were analyzed by Eurofins Environment Sweden AB (Linköping, Sweden) and Eurofins Vitamin Testing Denmark (Vejen, Denmark) as part of a storage trial implemented by CSIRO and Research Institutes of Sweden (RISE) that evaluated the micronutrient stability and sensory properties of fortified bouillon over time. Initial concentrations of the micronutrients were confirmed to be within acceptable ranges. The Ghana Food and Drugs Authority provided clearance for import and use of the bouillon cubes (FDA/FOD/FRD/FER/21/4158). Bouillon cubes were shipped to Ghana via air freight and were stored under refrigeration before distribution to households. After the completion of the acceptability trial (∼11 mo postproduction), micronutrient concentrations (vitamins A, B9, B12 and iodine) of the “upper-level” multiple micronutrient-fortified bouillon cubes (including both those stored under controlled conditions and those stored in ambient conditions in participants’ homes for 2 wk) were measured by Eurofins Vitamin Testing Denmark A/S (Vejen, Denmark).

### Participants

Women were recruited from 5 recruitment sites within the Kumbungu and Tolon districts (3 urban, 2 periurban/rural); households within the selected clusters were identified through a random walk method with door-to-door recruitment following a detailed sampling protocol.

Women were eligible to participate in the study if they were > 15 y of age and responsible for meal preparation in their households. If the household contained more than one potentially eligible woman, the head of household was asked to identify which of the eligible women best met the following additional criteria: woman regularly decides which meals will be prepared for the household and which ingredients will be used, and woman regularly prepares the main meal for the household. Exclusion criteria included: (*1)* severe illness warranting immediate hospital referral; (*2*) COVID-19 exposure, positive test, or current symptoms (including fever, cough, shortness of breath, loss of smell, vomiting, or diarrhea [> 3 liquid or semi-liquid stools in 24h]); (*3*) chronic severe medical condition (e.g. malignancy) or congenital anomalies requiring frequent medical attention or potentially interfering with nutritional status; *(4)* presence of ailments (such as toothache, or mouth pain) that may impact a participant’s ability to complete study activities; (*5)* pregnancy (self-reported), due to possible changes in food preferences and perceptions of organoleptic characteristics of foods; (*6*) inability to provide informed consent due to impaired decision making abilities; (*7*) current participation in a clinical trial; (*8)* reported shrimp, wheat, milk, soy, eggs, celery, fish, or mollusk allergy, or a previous adverse reaction to bouillon by anyone in the household; and (*9)* refusal to use study-provided bouillon cubes to prepare household meals while enrolled in the study.

The study was registered at clinicaltrials.gov (NCT05177614) and is reported according to the CONSORT reporting guidelines [25]. The study was approved by the Ghana Health Service Ethical Review Committee (GHS-ERC; 017/12/20) and the Institutional Review Board of the University of California, Davis (IRB; 1687671). Consent materials were presented, written (in English) and orally (in the local language, Dagbani) in the presence of an impartial witness. Informed consent was obtained from study participants prior to their enrollment and documented as either a written signature or a fingerprint. In the case of potentially eligible participants < 18 y of age, who were residing with their parent(s) or guardian(s), and who had never been married, divorced/separated, widowed, or lived together with someone, the participant’s parent or guardian provided written informed consent, and the participant provided assent.

### Study protocol

**FIGURE 1:**
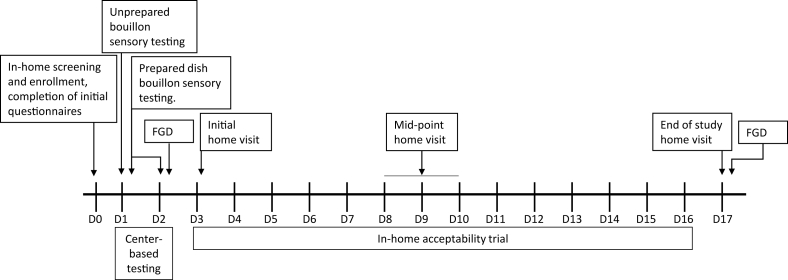
Timeline of study activities. Participants were enrolled in the study for 18 days; activities included 2 days of center-based sensory testing and a 2-wk in-home acceptability trial. FGD, focus group discussion.

Women were enrolled in the study for 18 d. See Figure 1 for a timeline of study activities.

### Structured interviews

On study day 0, detailed information was collected via structured interviews on the composition of the household, sociodemographic characteristics (e.g., education, household assets, water, hygiene and sanitation practices); food security (Household Food Insecurity Access Scale; HFIAS) [26]; household use of fortified foods (i.e., salt, wheat flour, cooking oil, and bouillon, based on a modified Fortification Assessment Coverage Toolkit; FACT) [27]; and social desirability, knowledge, attitudes and practices (KAP) regarding bouillon. A questionnaire to elicit hypothetical (i.e., stated) willingness-to-pay for fortified bouillon using a bidding-tree structure was also administered [28].

### Center-based sensory evaluations

Women reported to the testing center each morning for 2 consecutive days (study days 1 and 2). Participants were asked to not consume any foods for >1 h before their appointments. Each day, information about morbidity during the past 24h was recorded; women who reported possible infectious symptoms or ailments that might interfere with their ability to complete the study activities were excluded and referred for treatment, as appropriate. On study day 1, participants completed (1) acceptance and preference (affective) evaluations and (2) triangle (discrimination) tests on all 3 formulations of the dry bouillon cubes (i.e., bouillon cubes as purchased prior to cooking). On study days 1 and 2, participants completed acceptance and preference evaluations of 2 local dishes prepared with each of the bouillon cube formulations. Separate randomization schemes were computer-generated by the study statistician for each center-based evaluation.

#### Acceptance and preference evaluations of dry bouillon cubes:

Three unwrapped, unlabeled bouillon cubes were presented on a small tray to participants in random order. Using a 5-point hedonic scale (1 = dislike very much, 2 = dislike, 3 = neither like nor dislike, 4 = like, 5 = like very much) visualized using cartoon emotion faces, participants were asked to evaluate the acceptability of each of the 3 formulations of bouillon cubes for the following characteristics: appearance, feel (texture), crumble, smell (aroma), taste, and overall acceptability. Participants were asked about the presence of specific organoleptic characteristics of the cubes through “just-about-right” questions (e.g., color, hardness) and a “check all that apply (CATA)” questionnaire (e.g., metallic taste, aftertaste, too much or too little flavor, etc.) [29]. Participants’ additional opinions (positive or negative) about any specific bouillon cubes were documented. Finally, participants were asked to identify their most- and least-preferred cubes.

#### Triangle test of dry bouillon cubes:

Participants were presented with 3 unwrapped, unlabeled bouillon cubes, 2 of which (unknown to participant) were the same and one of which was different, and asked to identify the odd sample (visually, by touch, or by smell). This was repeated 3 times, with 3 different product pairings in a computer-generated random order [29]. Participants were also asked to describe in what way the samples differed (which sensory attributes) and if the observed difference was large, moderate, or uncertain.

#### Acceptance and preference evaluations of bouillon cubes in prepared dishes:

Participants completed acceptance and preference evaluations of 2 local dishes (dried okra soup and jollof rice) prepared with each of the 3 bouillon cube formulations using standardized recipes. These dishes were selected because they were commonly consumed by the study population (based on 24 h food lists collected during the pilot survey) and represented different food matrixes which could impact acceptability. Recipes were initially developed using quantitative data collected from in-home recipe observations completed as part of the pilot survey and further refined based on feedback from local enumerators (Supplementary Methods). One dish was provided per center-based testing day; the day on which each of the dishes was provided was randomized by cohort. Participants were given (sequentially, and in random order) small test portions (50.6 ± 0.6 g) prepared using each of the 3 types of bouillon cubes. Participants were asked to taste the first portion and eat as much as they wished within a 5-minute period; the amount of the test portion consumed was recorded (± 0.1 g) using an Ohaus CR221 scale (Parsippany, New Jersey). Participants were then asked to evaluate the acceptability of the dish for the following characteristics: appearance, color, smell (aroma), taste, aftertaste, saltiness, and overall acceptability. Acceptability was evaluated using the 5-point hedonic scale. “Just-about-right” questions and a “check all that apply” (CATA) questionnaire were used to solicit opinions about the presence of specific organoleptic characteristics of the dish. The aforementioned methods were repeated for the second and third portions. Participants were provided water and given a short rest period (∼1-2 min) between sampling each test preparation of the dish. After the participants evaluated all preparations of the dish, they were asked which preparation they preferred; additional opinions (positive or negative) were documented.

#### Two-week in-home household acceptability trial

Participants who completed the center-based sensory testing were individually randomized to receive 1 of the 3 bouillon cube formulations for the subsequent 2-wk in-home acceptability trial: (1) “upper-level” multiple micronutrient-fortified bouillon cube; (2) “lower-level” multiple micronutrient-fortified bouillon cube; or (3) control (fortified with iodine only) bouillon cube. The study statistician at the University of California, Davis prepared a computer-generated randomization scheme in blocks of 6. Three sealed, opaque envelopes bearing group allocations were presented to each participant, and the woman picked one to reveal her blinded study group assignment. To ensure blinding, study groups and their associated study-provided bouillon cubes were referred to by 3-digit alpha-numeric codes.

On the following day (study day 3), participants were visited in their homes by a study enumerator. Current stocks of commercial bouillon products were inventoried, and participants received a 2-wk ration of study-provided bouillon cubes. Participants, and their households, were instructed to not alter their typical cooking practices, but to simply substitute the study-provided bouillon cubes for the commercial bouillon cubes they typically used in all household cooking for the next 2 wk. Based on data from the pilot survey previously conducted in the same districts, household bouillon rations for the 2-wk in-home evaluation were set at approximately the 75^th^ percentile of household intake (g/d) (Table 2) [12]. Participants were requested to save all bouillon wrappers to facilitate monitoring of consumption. Participants received one midpoint visit (between study days 8-10) and an end-of-study visit on study day 17. At each visit, enumerators collected information about the number and types of bouillon products (both study-provided and nonstudy provided) that participants and other members of their households had used since the previous study visit, as assessed by questionnaire, observed stocks, and wrapper counts. At the midpoint visit, if a household had used more than one third of the bouillon cubes they had been provided, the enumerator provided the household with additional bouillon cubes to complete the in-home portion of the trial. Enumerators used structured interviews to elicit information about patterns of bouillon cube use (e.g., quantity typically used at each meal, addition of other condiments/salt), acceptability of the study-provided cubes (e.g., appearance, smell, taste, and overall acceptability) using the 5-point hedonic scale, and “just-about-right” and open-ended questions, and any morbidity symptoms of the participant or other household members in the previous week. On study day 17 only, enumerators also elicited information on perceived health effects of the bouillon cubes on household members (as reported by the index participant) and their endline hypothetical WTP for the bouillon cubes.

**TABLE 2:** Household bouillon rations provided to participants during the 2-wk in-home acceptability study

Total number of household members	Pilot survey apparent bouillon intake (g/d)^1^	Acceptability study, study-provided bouillon cubes, g/d	Number of 10 g bouillon cubes provided at baseline (2-wk ration)^2^
0 – 4	15.5 (9.8, 20.6)	25	40
5 – 9	16.7 (10, 20.6)	25	40
10 – 14	20 (12, 21.4)	25	40
15 – 19	20 (14.3, 25.6)	30	50
20 – 24	20 (20, 27.4)	30	50
25+	33.3 (21.4, 60)	50	80

1 Data are from the pilot survey previously conducted in the same districts [12]. Value median (IQR).

2 Bouillon rations were set at approximately the 75^th^ percentile of reported intake from the pilot survey conducted in the same districts [12].

### Focus group discussions

Focus group discussions (*n =* 10) were conducted among all study participants after the center-based sensory testing (day 2), and again at the end of the in-home portion of the acceptability study (day 17); women participated in one or the other, but not both days of focus group discussions (*n =* 5-9 participants per focus group discussion). On study day 2, we elicited perspectives on the sensory attributes of the study-provided bouillon cubes, how they compared to commercial bouillon products, and how the participants anticipated using the study-provided bouillon cubes in their households during the in-home portion of the trial. On study day 17 (endpoint), we elicited similar information, as well as their perspectives on use of the study-provided bouillon cubes in their households, and willingness to continue use of the study-provided bouillon cubes. Focus group discussions were conducted by a trained facilitator in the local language (Dagbani) following a semi-structured interview guide; a trained note taker was also present to systematically gather participant responses and reactions during the focus group discussions. All focus group discussions were audio-recorded.

### Sample size estimation

The sample size for this study was estimated on the basis of the ability to detect a moderate effect size difference of 0.52 SD in the acceptability of the multiple-micronutrient fortified bouillon cubes in a three-way comparison of the study products (α = 0.05; β = 0.20) [30]. Based on these estimates, 75 women were required to complete the center-based portion of the study. For the in-home portion of the acceptability trial, this sample size (*n =* 25 women per study group) would allow us to detect an 0.78 SD effect size difference in three-way comparisons on continuous measures of acceptability and consumption. To allow for possible attrition, the sample size was increased by ∼10% to 84 women.

### Statistical analysis

A detailed statistical analysis plan was developed prior to analysis and is available online [19]. Descriptive statistics were calculated for all variables and relevant model assumptions were assessed (e.g., normality and homoscedasticity of residuals using Shapiro-Wilk and Breusch-Pagan tests). Estimated apparent daily intake of bouillon by individuals was calculated using the adult male equivalent (AME) method. We assumed that food allocation (including bouillon) within a household was in proportion to each members’ energy requirements. Energy requirements of an 18–30-y-old male served as the reference value (AME = 1), all other age and sex groups were weighted relative to the adult male based on their respective energy requirements (e.g., non-pregnant, non-lactating female 18–30 y old = 0.787; female child 2–3 y old = 0.344), and total household AMEs were calculated. Individual household members’ estimated apparent daily intake of bouillon (g/d) was calculated as follows: individual AME/total household AMEs x apparent household intake of bouillon (g/d) [31, 32]. Estimated apparent daily intake of bouillon is presented for women of reproductive age and children 2–5 y old separately, as these are the target groups for the planned randomized controlled efficacy trial. Outcomes based on the 5-point hedonic scale were analyzed as both continuous and dichotomous outcomes. For dichotomous outcomes, participants’ responses were re-classified into categories of “liked” (scores of 4 or 5) and “not-liked” (scores of 1-3). Just-about-right outcomes were analyzed as dichotomous variables by reclassifying participants into either ‘right’ (score of just-about-right) compared with ‘not right’ (score of either too much or too little). Continuous outcomes (e.g., 5-point hedonic scales, apparent bouillon intake, hypothetical WTP, etc.) were analyzed using ANOVA models followed by post-hoc pairwise comparisons to estimate mean differences if the outcome was found to differ across the 3 study groups. Dichotomous outcomes (e.g., liked compared with not-liked, just-about-right, organoleptic properties, health effects, reported symptoms, etc.) were analyzed using modified Poisson regression [33], followed by post-hoc pairwise comparisons to estimate prevalence ratios if the outcome was found to differ across the 3 study groups. Triangle test outcomes were analyzed using a binomial probability approach where the expected proportion of correct answers is 1/3 [34]. Dependence in observations was accounted for by incorporating robust standard errors with participant as the independent unit of randomization. In addition, ‘study day’ and ‘order served’ were controlled for in regression models for outcomes where the sequence of administration was randomized.

Data were analyzed in R version 4.1.1 (R Core Team, Vienna, Austria). Data are presented as means + SD or median (IQR), unless otherwise noted. All testing and confidence intervals are 2-sided, confidence intervals are 95% and a *P*-value < 0.05 is considered statistically significant. The investigators remained blinded to treatment groups until all primary analyses were completed.

Focus group discussion recordings were translated to English and transcribed independently by 2 members of the study-team (one of whom was KWN), not involved in the focus group discussions. Transcripts were reviewed by KRW for consistency with quantitative findings, and exemplar quotes were selected through discussion with coauthors.

## Results

**FIGURE 2:**
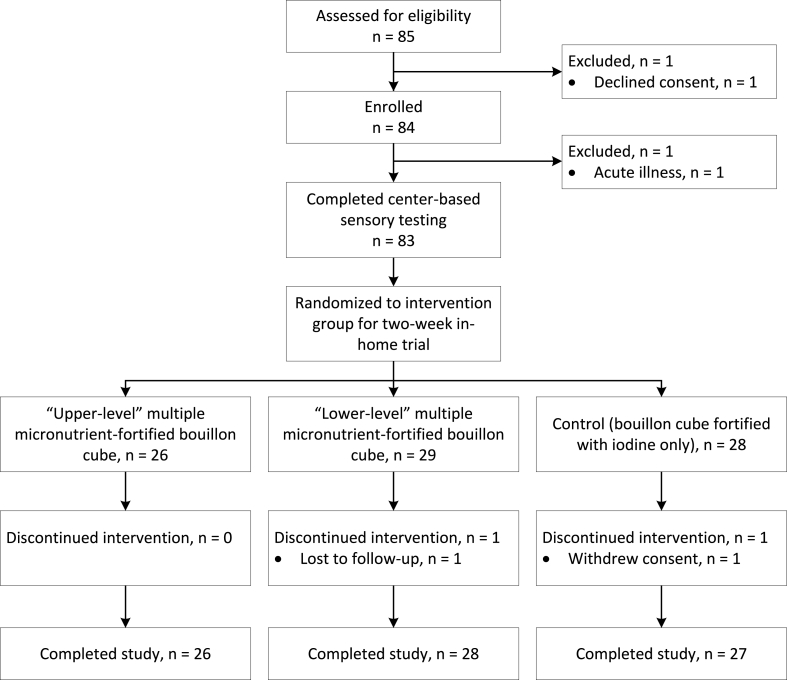
Flowchart of participant progression through the acceptability study.

Eighty-five households were approached, and women were screened for eligibility. Eighty-four participants (99%) were enrolled, 83 participants completed the center-based evaluation of bouillon cubes, and 81 participants (96% of those enrolled) completed the study through day 17 (Figure 2). Demographic and socio-economic characteristics of individual participants and their households are presented in Table 3 [35,36].

**TABLE 3:** Demographic and socio-economic characteristics of households and individual study participants (*n =* 84)

Variable	Value^1^
Individual characteristics	
Age, y	37.9 + 13.3
Household characteristics^2^	
Household size	13.8 + 8.7
Education of head of household	
None	68 (81.0)
Primary	6 (7.1)
Secondary or higher	10 (11.9)
Food insecurity, moderate or severe	53 (63.1)
Household water source	
Surface water	44 (52.4)
Unimproved	0 (0)
Improved	40 (47.6)
Household sanitation facility	
Open defecation (no facility)	62 (73.8)
Unimproved	4 (4.8)
Improved	18 (21.4)
Electricity in household	75 (89.3)
Mobile phone owned by at least one member of household	78 (92.9)

1 Value mean + SD or n (%).

2 Food security assessed using the Household Food Insecurity Access Scale (HFIAS) [26]; Household water source and sanitation facility defined using the WHO UNICEF Joint Monitoring Programme ladders [45, 46]. Improved sanitation facilities are those designed to hygienically separate excreta from human contact and include flush/pour flush toilets and pit latrines with slabs (including ventilated pit latrines). Unimproved sanitation facilities include pit latrines without a slab or platform.

Study participants’ knowledge, attitudes, and practices with regards to bouillon are presented in Table 4. 92.9% of participants (*n =* 78/84) reported cooking with bouillon at least twice per day, and apparent intake of bouillon was 2.29 (1.29, 3.90) g/AME/d. Households primarily chose the brand and flavor of bouillon cubes based on taste (94.0%, *n =* 79/84), family preference (52.4%, *n =* 44/84), and availability (29.8%, *n =* 25/84). The majority of participants (63.1%, *n =* 53/84) thought having bouillon in their diets was good, with common reasons cited that it improves health, improves the taste of food, and encourages eating. 19% of participants (*n =* 16/84) considered having bouillon in their diets to be bad, with common reasons being that it caused joint or “waist” (i.e., abdominal or back) pain and high blood pressure. 11% of participants (*n =* 9/84) had ever heard of fortified bouillon, and most considered it good for their health (*n =* 8/84); however, only 4% of participants (*n =* 3/84) had ever knowingly purchased fortified bouillon.

**TABLE 4:** Apparent household intake of bouillon, and knowledge, attitudes, and practices of study participants^1^ (*n* = 84)

Variable	Value^2^
Apparent household intake of bouillon^3^	
Apparent household intake of bouillon, g/d	19.7 (11.0, 25.2)
Apparent intake of bouillon by individuals, g/capita/d	1.68 (0.90, 2.86)
Apparent intake of bouillon by individuals, g/AME/d	2.29 (1.29, 3.90)
Apparent intake of bouillon by WRA, g/d	2.03 (1.05, 3.20)
Apparent intake of bouillon by children 2-5y, g/d	0.83 (0.47, 1.37)
Bouillon knowledge, attitudes, and practices	
Household has ever cooked with bouillon	84 (100)
How often do you cook with bouillon?	
At least twice per day	78 (92.9)
Once per day	5 (6.0)
4 – 6 times per week	1 (1.2)
Flavor of bouillon cooked with most often	
Shrimp	83 (98.8)
Other (beef, chicken, vegetable, etc.)	1 (1.2)
Type of bouillon cooked with most often^4^	
10 g cube	39 (46.4)
12 g cube	45 (53.6)
Other (powder, liquid)	0 (0)
Frequency of bouillon purchasing (interval of days between purchasing)	21 (7, 30)
Person responsible for providing money to purchase bouillon	
Head of household	27 (32.1)
Study participant	55 (65.5)
Other	2 (2.4)
To what extent is it up to you to add bouillon to the dishes that you typically cook with bouillon?	
Completely or mostly up to me	52 (61.9)
Both up to others and up to me	32 (38.1)
Completely or mostly up to others	0 (0)

1 AME, adult male equivalent. WRA, women of reproductive age.

2 Values median (IQR) or n (%).

3 Household intake and apparent intake estimated using a 30-day household questionnaire, based on the Fortification Assessment Coverage Tool [27]. Apparent intake of bouillon by WRA and children 2-5y (g/d) was calculated using the AME method as follows: (age/sex-specific AME)/(total household AME) x apparent household intake of bouillon (g/d).

4 Commercial bouillon cubes are available as either 10 g or 12 g cubes, depending on the producer. Bouillon cubes provided by the present acceptability study were 10 g.

### Center-based sensory evaluations

#### Acceptance and preference evaluations of bouillon cubes

There were no statistically significant differences in the mean overall liking among the 3 formulations when the bouillon was presented dry or prepared in either of 2 recipes (dried okra soup and jollof rice) (Table 5). When analyzed as a dichotomous outcome (i.e., “liked” compared with “not-liked”), > 89% of participants rated their overall liking of the 3 bouillon product formulations as “like” or “like very much”, across evaluations of both the unprepared cubes, and the cubes in local recipe preparations. However, fewer participants rated their overall liking of the “upper-level” multiple micronutrient-fortified bouillon cube as “like” or “like very much” (89.2%, *n =* 74/83) compared with the control cube (98.8%, *n =* 82/83) when prepared in dried okra soup (*P* = 0.013); there were no differences for the dry bouillon or the jollof rice (Supplemental Table 1). Acceptability of the bouillon cubes’ appearance, color, feel, aroma, taste, saltiness, and aftertaste did not differ among the 3 formulations; however, the crumble of the “upper-level” multiple micronutrient-fortified bouillon cube was less well liked compared with the other 2 formulations (Table 5; Supplemental Table 1). The majority of participants (50.6 – 89.2%, *n =* 42/83 – 74/83) rated the bouillon cubes as “just-about-right” for hardness, color (pink), texture, stickiness, saltiness and aftertaste, and there were minimal differences across the formulations (Supplemental Table 2). Fewer than 5% of participants (*n* < 5/83) negatively characterized the bouillon cubes as having a metallic, bland, rancid, or bitter taste, and these characterizations also did not differ across formulations (Supplemental Table 3). In preference testing, significantly more participants reported preferring the control bouillon cube (48.2%, *n =* 40/83) compared with the “lower-level” multiple micronutrient-fortified bouillon cube (18.1%, *n =* 15/83) when evaluating the dry bouillon cubes (*P* = 0.003; Table 5); however, 33.7% of participants (*n =* 28/83) preferred the “upper-level” micronutrient-fortified bouillon cube, and this did not differ significantly from either of the other 2 formulations. There were no differences in preference for the different bouillon cube formulations in the prepared dishes.

**TABLE 5:** Center-based evaluations of acceptability and preference among different formulations of multiple micronutrient-fortified bouillon cubes

Variable	Bouillon cube formulation^1^			
	“Upper-level”	“Lower-level”	Control	*P*-value^2^
Participants, n	83	83	83	
Acceptability^3^				
Dry bouillon				
Overall liking	4.3 ± 1.0	4.5 ± 0.6	4.5 ± 0.8	0.17
Appearance	4.3 ± 0.8	4.2 ± 0.8	4.4 ± 0.7	0.12
Feel	4.2 ± 0.8	4.4 ± 0.7	4.4 ± 0.7	0.16
Crumble	4.1 ± 0.9^b^	4.4 ± 0.7^a^	4.4 ± 0.7^a^	0.03
Aroma	4.3 ± 1.0	4.3 ± 0.9	4.3 ± 1.0	0.86
Taste	4.3 ± 0.9	4.3 ± 0.8	4.3 ± 0.7	0.80
Prepared bouillon (dried okra soup)				
Overall liking	4.3 ± 1.0	4.5 ± 0.8	4.6 ± 0.6	0.08
Appearance	4.5 ± 0.8	4.4 ± 0.7	4.5 ± 0.6	0.32
Color	4.5 ± 0.7	4.3 ± 0.8	4.5 ± 0.7	0.19
Aroma	4.5 ± 0.9	4.4 ± 0.7	4.5 ± 0.7	0.48
Taste	4.4 ± 0.9	4.4 ± 0.8	4.6 ± 0.8	0.09
Saltiness	4.3 ± 0.9	4.5 ± 0.8	4.3 ± 0.9	0.27
Aftertaste	4.1 ± 1.0	4.2 ± 0.8	4.3 ± 0.8	0.43
Prepared bouillon (jollof rice)				
Overall liking	4.4 ± 0.7	4.4 ± 0.8	4.3 ± 0.9	0.36
Appearance	4.6 ± 0.7	4.4 ± 0.7	4.4 ± 0.8	0.06
Color	4.6 ± 0.6	4.4 ± 0.7	4.4 ± 0.7	0.13
Aroma	4.6 ± 0.7	4.5 ± 0.6	4.4 ± 0.8	0.07
Taste	4.6 ± 0.7	4.5 ± 0.8	4.4 ± 0.7	0.28
Saltiness	4.3 ± 0.7	4.4 ± 0.9	4.4 ± 0.9	0.96
Aftertaste	4.2 ± 0.9	4.2 ± 0.8	4.1 ± 0.8	0.64
Preference^4^				
Dry bouillon	28 (33.7)^ab^	15 (18.1)^a^	40 (48.2)^b^	0.003
Prepared bouillon (dried okra soup)	29 (34.9)	29 (34.9)	25 (30.1)	0.96
Prepared bouillon (jollof rice)	27 (32.5)	29 (34.9)	27 (32.5)	0.87
Percentage of test meals consumed				
Prepared bouillon (dried okra soup)	25.0 ± 27.8	27.0 ± 26.7	25.6 ± 26.2	0.51
Prepared bouillon (jollof rice)	33.2 ± 24.2	32.8 ± 24.1	32.0 ± 24.4	0.68

1 Value mean ± SD or n (%). “Upper-level” multiple micronutrient fortified bouillon cubes were fortified with vitamin A (200 μg RE/g), folic acid (80 μg/g), vitamin B12 (1.2 μg/g), iron (4 mg/g), zinc (3 mg/g), and iodine (30 μg/g). “Lower-level” multiple micronutrient fortified bouillon cubes were fortified with vitamin A (96 μg RE/g), folic acid (28.8 μg/g), vitamin B12 (0.288 μg/g), iron (1.3 mg/g), zinc (1.68 mg/g), and iodine (30 μg/g). The control bouillon cube was fortified with iodine (30 μg/g) only.

2 *P*-values from ANOVA models followed by post-hoc pairwise comparisons, indicated by superscripts, if the outcome was found to differ.

3 Overall liking and acceptability of various organoleptic characteristics were assessed using a 5-point Likert scale: 1 = Dislike very much, 2 = Dislike, 3 = Neither like nor dislike, 4 = Like, 5 = Like very much.

4 n (%) indicating each cube as their most-preferred formulation.

Focus group discussions largely corroborated these findings. As one participant stated “*The color, appearance, taste, and everything about [the bouillon] were okay for us. None of those we tasted today failed. We are grateful for its color, appearance, and everything about it. They were all perfect for us*” (cohort 5, participant 3). However, focus group discussion participants did note differences among the bouillon cube formulations, characterizing one of the cubes as being “saltier” (less preferred) and one being “fishier” (preferred), even though these differences were not readily apparent in the hedonic acceptability testing. Participants stated, “*What I liked about [the bouillon] was the one that had a lot of fish in it, that was the one that we all liked*” (cohort 1, participant 2), and “*you can taste one [bouillon] and it seems salty, so for that one, you should reduce the salt content*” (cohort 2, participant 120). However, all participants expressed willingness to exclusively use the study-provided bouillon cubes during the 2 wk of the in-home portion of the acceptability trial. As one participant stated, “*we will want to cook with them because we enjoyed it yesterday. We will always use it in the event that you bring it out*” (cohort 5, participant 4). Other participants mentioned the perceived health benefits of a micronutrient-fortified bouillon cube in addition to the acceptability. As one participant noted “*I will take it home to cook with it because you told us you have fortified it with nutrients, and everyone wants to be healthy. That is why we will want to take it home*” (cohort 1, participant 6).

#### Triangle test of dry bouillon cubes

Participants were able to correctly differentiate among the 3 cubes in discrimination testing. 86.7% of participants (*n =* 72/83) could correctly identify which bouillon cube sample differed from the other 2 comparing between “upper-level” multiple micronutrient-fortified and control bouillon cubes (*P* < 0.001; probability of choosing the correct sample by chance was 33.3%). Similarly, 78.3% (*n =* 65/83) and 68.7% (*n =* 57/83) of participants were able to correctly identify between the “lower-level” multiple micronutrient-fortified and control bouillon cubes, and the “lower-level” and “upper-level” multiple micronutrient-fortified bouillon cubes, respectively (*P* < 0.001). Participants most commonly reported being able to discriminate among cubes based on color (84.3–94.0% of comparisons, *n* = 70/83 to 78/83), smell (47.0–50.6%, *n* = 39/83 to 42/83), crumble (34.9–47.0%, *n* = 29/83 to 39/83) and hardness (39.8–43.4%, *n* = 33/83 to 36/83). For example, one focus group participant noted “*in terms of appearance some were white…and there were some whose color was a bit more pronounced*” (cohort 1, participant 6). Another participant stated “*[one bouillon] was hard and a little rough, but the other 2 were smooth*” (cohort 1, participant 3).

### In-home household acceptability trial

#### Acceptance and preference evaluations of bouillon cubes

After the 2-wk in-home trial, there were no significant differences in the mean overall liking among the 3 formulations of bouillon (Table 6). 93.8% of index participants (*n =* 75/80) rated their overall liking of the bouillon product formulation to which they were randomly assigned as “like” (4) or “like very much” (5), and this did not differ among groups (*P* = 0.91; Supplemental Table 4). Similarly, 92.5% of the participants (*n =* 74/80) reported that the bouillon cubes were well liked by their household members (“like” (4) or “like very much” (5), *P* = 0.91). The acceptability of specific organoleptic properties (e.g., appearance, aroma, taste, crumble, etc.) did not differ among the 3 formulations (Table 6; Supplemental Table 4)**.** The majority of participants (67.5 – 93.8%, *n =* 54/80 – 75/80) rated the bouillon cubes as “just-about-right” for hardness, color (pink), texture, stickiness, saltiness and aftertaste, and there were no differences across the formulations (Supplemental Table 5). No study participants negatively characterized the bouillon cubes as having a metallic, bland, rancid, or bitter taste (Supplemental Table 6).

**TABLE 6:** In-home evaluations at endline of acceptability among different formulations of multiple micronutrient-fortified bouillon cubes

Variable	Bouillon cube formulation^1^			
	“Upper-level”	“Lower-level”	Control	*P*-value^2^
Participants^3^, n	26	27	27	
Overall acceptability				
Index participant	4.5 ± 0.7	4.4 ± 0.7	4.5 ± 0.5	0.91
Household members	4.3 ± 0.9	4.4 ± 0.6	4.5 ± 0.5	0.52
Dry bouillon 4				
Appearance	4.5 ± 0.5	4.6 ± 0.5	4.6 ± 0.5	0.90
Feel	4.3 ± 0.5	4.4 ± 0.5	4.3 ± 0.4	0.52
Crumble	4.5 ± 0.5	4.4 ± 0.5	4.5 ± 0.5	0.80
Aroma	4.2 ± 0.8	4.3 ± 0.7	4.2 ± 0.8	0.94
Taste	4.5 ± 0.5	4.5 ± 0.5	4.6 ± 0.5	0.86
Prepared bouillon (household dishes)				
Appearance	4.3 ± 0.8	4.4 ± 0.6	4.4 ± 0.6	0.82
Color	4.3 ± 0.9	4.3 ± 0.8	4.3 ± 0.7	0.94
Aroma	4.4 ± 0.7	4.4 ± 0.6	4.6 ± 0.6	0.72
Taste	4.6 ± 0.5	4.6 ± 0.5	4.6 ± 0.5	0.96
Saltiness	4.3 ± 0.5	4.4 ± 0.5	4.4 ± 0.5	0.76
Aftertaste	4.2 ± 0.5	4.3 ± 0.6	4.4 ± 0.7	0.34

1 Value mean ± SD. “Upper-level” multiple micronutrient fortified bouillon cubes were fortified with vitamin A (200 μg RE/g), folic acid (80 μg/g), vitamin B12 (1.2 μg/g), iron (4 mg/g), zinc (3 mg/g), and iodine (30 μg/g). “Lower-level” multiple micronutrient fortified bouillon cubes were fortified with vitamin A (96 μg RE/g), folic acid (28.8 μg/g), vitamin B12 (0.288 μg/g), iron (1.3 mg/g), zinc (1.68 mg/g), and iodine (30 μg/g). The control bouillon cube was fortified with iodine (30 μg/g) only.

2 P-values from ANOVA models followed by post-hoc pairwise comparisons, indicated by superscripts, if the outcome was found to differ.

3 Data unavailable for one participant in the “lower-level” bouillon cube formulation group.

4 Overall acceptability and acceptability of various organoleptic characteristics were assessed using a 5-point Likert scale: 1 = Dislike very much, 2 = Dislike, 3 = Neither like nor dislike, 4 = Like, 5 = Like very much.

The focus group discussions conducted after the in-home acceptability portion of the trial confirmed that the different bouillon formulations were generally well accepted by the study participants. As one participant stated, “*its appearance, aroma, how it feels in the hand, and everything about it is just right as how bouillon ought to be. It has no bad scent…Everything about it is just like how every food of a human being ought to be, so it has nothing that shows it is not good*” (cohort 2, participant 3). Although a few participants mentioned color changes to their soups, these reports were distributed among the 3 bouillon formulations. One participant mentioned, “*I like its aroma and taste, but as for the color...when you use 2 cubes to cook, it will change the soup and even if the soup stays overnight, the appearance of the soup will [darken]”* (cohort 2, participant 2).

#### Apparent intake of study-provided bouillon cubes

Median apparent intake of study-provided bouillon was 3.6 g/capita/d and did not differ by study group (Table 7). Based on the AME method, the median apparent bouillon intake of women of reproductive age ranged from 3.9 to 5.0 g/d, and that of children 2 to 5 y ranged from 1.8 to 2.4 g/d. 79.3% (*n* = 65/82) of households received additional bouillon at the midpoint visit to ensure adequate quantities for the duration of the in-home portion of the study. During focus group discussions, reported changes in bouillon intake during the in-home acceptability portion of the trial (compared with the weeks preceding the trial) were variable. Many participants reported increasing the number of bouillon cubes used during the in-home portion of the acceptability trial. As one participant stated “*I use 2, but with the old one, I used one because for this one even if you use 4, you can still eat, it is not sweet*^1^*, it is tasty. I want its taste and because of that I add more into soup*” (cohort 2, participant 3). However, according to one study participant, whose opinion was echoed by others, “*we did not change anything in cooking; we cooked the same way we used to cook before you gave them to us. If you usually cook with one [bouillon] cube, that is how you will be cooking, and if it is 2 cubes, that is what you will be cooking with so for that matter we did not change anything*” (cohort 1, participant 3). Reported sharing of study-provided bouillon cubes with others outside of the household was rare (*n =* 2/80); several households reported using the study-provided bouillon cubes in extra-household cooking when hosting an event during the trial (e.g., naming ceremony), or when preparing food for nonhousehold members (e.g., apprentices, laborers, elderly neighbors).

**TABLE 7:** Estimated apparent intake of different formulations of multiple micronutrient-fortified bouillon cubes over the 2-wk in-home acceptability trial period^1^

Variable	Bouillon cube formulation^2^			
	“Upper-level”	“Lower-level”	Control	*P*-value^3^
Participants, n	26	28	27	
Two-week estimate of apparent bouillon intake				
Apparent household intake of bouillon, g/d	42.9 (30.7, 65.0)	43.6 (35.0, 60.0)	42.9 (32.1, 81.4)	0.50
Apparent household intake of bouillon, g/capita/d	4.1 (3.0, 5.8)	3.6 (2.6, 4.8)	3.3 (2.5, 6.3)	0.91
Apparent household intake of bouillon, g/AME/d	5.5 (4.4, 8.9)	5.0 (3.8, 7.0)	4.9 (3.6, 8.0)	0.72
Apparent intake of bouillon by WRA^4^, g/d	5.0 (3.5, 7.0)	3.9 (3.2, 6.1)	4.3 (2.7, 6.2)	0.50
Apparent intake of bouillon by children 2-5y^4^, g/d	2.4 (1.9, 3.6)	1.8 (1.2, 2.5)	2.1 (1.5, 3.3)	0.59

1 AME, adult male equivalent; WRA, women of reproductive age.

2 Value median (IQR). “Upper-level” multiple micronutrient fortified bouillon cubes were fortified with vitamin A (200 μg RE/g), folic acid (80 μg/g), vitamin B12 (1.2 μg/g), iron (4 mg/g), zinc (3 mg/g), and iodine (30 μg/g). “Lower-level” multiple micronutrient fortified bouillon cubes were fortified with vitamin A (96 μg RE/g), folic acid (28.8 μg/g), vitamin B12 (0.288 μg/g), iron (1.3 mg/g), zinc (1.68 mg/g), and iodine (30 μg/g). The control bouillon cube was fortified with iodine (30 μg/g) only.

3 *P* values from modified Poisson regression.

4 Apparent intake of bouillon by WRA and children 2-5y (g/d) was calculated using the AME method as follows: (age/sex-specific AME)/(total household AME) x apparent household intake of bouillon (g/d).

A total of 40.0% and 31.3% of participants reported perceived positive health effects of consuming the study-provided bouillon cubes among themselves (*n =* 32/80) and their household members (*n =* 25/80), respectively, and this did not differ by study group (*P* = 0.96, *P* = 0.54). The most reported positive perceptions of health outcomes were that the study-provided bouillon cubes “improved health” and “encouraged eating” (data not shown). Reported perceived negative health effects of consuming the study-provided bouillon cubes were rare, both among index participants (*n =* 1/80) and their household members (*n =* 2/80), with “upset stomach” being the most reported negative effect. During the focus group discussions, participants reported initial concerns with using the study provided bouillon cubes. According to one participant, “*members of our household thought we will be sick if we consume it. But when we consumed it none of us experienced any problems regarding it”* (cohort 1, participant 2). Many focus group participants ascribed health benefits to the study-provided bouillon. One participant stated, “*from our observation, we have concluded that if we continue to consume this [bouillon], we will experience [better] health in our lives than the [bouillon] that we have been consuming*" (cohort 2, participant 3).

### Hypothetical willingness-to-pay

On day 0 (i.e., before participants had been exposed to the study cubes), hypothetical WTP for a single, 10-g bouillon cube that was described as being fortified with multiple micronutrients ranged from 0.32 to 0.40 Ghana cedis (GH₵), or US $0.05 to 0.07 using the average exchange rate from December 2021 to January 2022 of 6.065 Ghana cedis to the US dollar, and did not differ among study groups (*P* = 0.67) (Table 8). At endline, average hypothetical WTP for the 10-gram study cube, which was elicited from participants without mentioning that the study cube may have been fortified with multiple micronutrients, ranged from GH₵ 0.23 to 0.27 (US $0.037–0.04) and did not differ among groups (*P* = 0.64). When participants were asked their hypothetical WTP for the study cube *if* it were fortified with multiple micronutrients, average hypothetical WTP ranged from GH₵ 0.23 to 0.28 (US $0.037–0.05) and did not differ among groups (*P* = 0.47). There was no difference in hypothetical WTP for the study cube if it were fortified with multiple micronutrients, compared with no mention of fortification, either among groups (*P* = 0.46) or within participants (*P* = 0.12). Finally, hypothetical WTP for a multiple-micronutrient fortified bouillon cube was higher at day 0 than at endline (*P* = 0.03), but this difference did not vary among study groups (*P* = 0.44).

**TABLE 8:** Hypothetical willingness-to-pay for multiple micronutrient-fortified bouillon cubes^1^

Variable	Bouillon cube formulation^2^			
	“Upper-level”	“Lower-level”	Control	*P*-value^3^
Participants, n	20	21	23	
Day 0 hypothetical WTP for multiple micronutrient-fortified bouillon cubes, cedis (GH₵)/10 g cube	0.32 ± 0.23	0.40 ± 0.39	0.35 ± 0.26	0.67
Endline hypothetical WTP for study cubes, cedis (GH₵)/10 g cube	0.27 ± 0.20	0.23 ± 0.08	0.26 ± 0.10	0.64
Endline hypothetical WTP for study cubes if multiple micronutrient-fortified, cedis (GH₵)/10 g cube	0.28 ± 0.20	0.23 ± 0.08	0.27 ± 0.11	0.47
Difference in endline hypothetical WTP for study cube and study cube if multiple micronutrient-fortified^4^, cedis (GH₵)/10 g cube	0.01 ± 0.03	0.0 ± 0.0	0.02 ± 0.06	0.46^3^, 0.12^5^
Difference in day 0 and endline hypothetical WTP for multiple micronutrient-fortified cube^6^, cedis (GH₵)/10 g cube	0.04 ± 0.32	0.17 ± 0.40	0.07 ± 0.29	0.44^3^, 0.03^5^

1 WTP, willingness-to-pay.

2 Values mean ± SD. All values reported in Ghana cedis (GH₵) per 10 g cube. “Upper-level” multiple micronutrient fortified bouillon cubes were fortified with vitamin A (200 μg RE/g), folic acid (80 μg/g), vitamin B12 (1.2 μg/g), iron (4 mg/g), zinc (3 mg/g), and iodine (30 μg/g). “Lower-level” multiple micronutrient fortified bouillon cubes were fortified with vitamin A (96 μg RE/g), folic acid (28.8 μg/g), vitamin B12 (0.288 μg/g), iron (1.3 mg/g), zinc (1.68 mg/g), and iodine (30 μg/g). The control bouillon cube was fortified with iodine (30 μg/g) only.

3 *P* values for difference across the study groups based on ANOVA model.

4 Difference calculated as hypothetical WTP for multiple micronutrient-fortified study bouillon cubes minus hypothetical WTP for study cube.

5 *P* value for pooled difference between baseline and endline in mean WTP based on t-test.

6 Difference calculated as day 0 WTP for multiple micronutrient-fortified study bouillon cube minus endline WTP for multiple micronutrient-fortified study bouillon cube.

#### Post-household micronutrient analysis of bouillon cubes

Bouillon cubes analyzed after the completion of the acceptability trial, including those stored under controlled conditions, and those stored under ambient conditions in the households for 2 wk, retained micronutrient concentrations within the expected ranges (Supplemental Table 7).

## Discussion

Results from this multiple micronutrient-fortified bouillon cube acceptability study indicate that the 3 bouillon cube formulations, including those containing “lower” and “upper” levels of multiple micronutrients and a control cube fortified with iodine only, were well accepted by participants and their households, during both the center-based sensory testing and the 2-wk in-home portion of the study. Although there were some suggestions that the “upper-level” multiple micronutrient fortified bouillon cubes were slightly less well liked than the control, sensory testing did not identify consistent differences in the participants’ evaluations of the overall acceptability of the different bouillon cube formulations, nor in their evaluations of the specific organoleptic characteristics of each formulation. In addition, apparent bouillon intake did not differ among the 3 bouillon formulations during the in-home portion of the study, and in all groups, the average quantity of cubes consumed by the household was greater than what they typically reported purchasing. Finally, after the 2-wk in-home trial period, stated hypothetical WTP for the study cubes did not differ among groups. Thus, multiple micronutrient-fortified and control bouillon cubes appear to be acceptable to women and their households in these 2 districts in northern Ghana.

To our knowledge, this is the first study reporting acceptability data for multiple micronutrient-fortified bouillon. Although some multinational food companies are currently voluntarily fortifying bouillon cubes with iron or vitamin A (in addition to iodine), micronutrient concentrations of selected micronutrients in these commercially available cubes are 75 to 85% lower than those tested in the present study [8, 13, 14]. Results of the present study indicate that fortifying bouillon cubes with multiple micronutrients at higher concentrations is acceptable for a clinical trial designed to evaluate the efficacy of multiple micronutrient-fortified bouillon cubes for improving micronutrient status.

The iodization of salt and salt-containing condiments, such as bouillon, has been shown to be a successful strategy in the control and elimination of iodine deficiency disorders worldwide [37]. Expanding the range of micronutrients carried by salt and other condiments represents a novel way to increase nutritional adequacy among individuals at high risk of inadequate micronutrient intakes and micronutrient deficiencies [7]. Thus, condiments have received renewed attention as fortification vehicles [9]; however, the evidence regarding technical feasibility remains limited. A challenge in the development of fortified foods, including fortified condiments, has been maintaining the organoleptic attributes of the unfortified product. Historically, there have been numerous sensory challenges associated with the addition of iron to fortified products (e.g., double-fortified salt), including discoloration of the fortified product, degradation of iron encapsulation, the appearance of black particles in prepared foods, or the darkening of foods with cooking [38]. In the present study, we used ferric pyrophosphate, which is a less reactive and whiter iron compound compared to other common forms, such as ferrous sulfate [39, 40]. In the present study, discrimination testing revealed that participants could distinguish among the different bouillon cube formulations, with the majority of participants citing color differences as the organoleptic characteristic, which allowed them to differentiate among cubes. Color differences were likely related to differences in the amount and make-up of the premix added to the “upper-level” and “lower-level” multiple micronutrient-fortified cubes (i.e., formulations contained differing concentrations of micronutrients and citric acid/trisodium citrate enhancers) compared with the control cubes fortified with iodine only. However, we note that there is wide variation in color among different brands of commercially available cubes and that this variation by brand is greater than color differences among the study bouillon formulations with and without multiple micronutrients (Supplemental Figure 1). In addition, there were no differences in the *acceptability* of the appearance, color, smell, or taste among the different bouillon formulations and no differences in reported negative organoleptic attributes (e.g., ‘metallic’ or ‘off-taste’). The addition of premix may have also affected the crumble and texture (i.e., smoothness, fineness of the participles) of the cube, which were rated lower in the “upper-level” micronutrient-fortified bouillon cubes (vs. the control bouillon cubes) in the center-based acceptability portion of the trial. This possibility is consistent with participant reports that they distinguished among cubes based on crumble (38–51%) and hardness (40–47%). Although there were a few qualitative reports of the study-provided bouillon cubes “darkening” specific soups, these were distributed among the 3 bouillon cube formulations, and there were no differences in ratings of any organoleptic characteristics or bouillon consumption among the groups during the 2-wk in-home portion of the trial, although the sample size was small (*n* = 26 - 28 per group). Thus, although participants were able to distinguish among cubes when presented side by side, there was not a clear negative impact on specific organoleptic characteristics when used in the context of daily cooking.

During the 2-wk in-home portion of the acceptability trial, apparent household intake of bouillon was greater than predicted from baseline assessments of apparent bouillon consumption among participants in this study, as well as in a similar population assessed during the pilot survey conducted 1 y prior [12]. Although we cannot directly compare differences in apparent bouillon intakes estimated prior to and during the acceptability trial due to differences in assessment methods (i.e., FACT household questionnaire vs. estimation based on wrapper counts), nor fully understand the reasons for this apparent increase (e.g., increase in bouillon consumption due to free distribution of bouillon vs. increased inter-household sharing of bouillon), these findings suggest that further research to evaluate the efficacy of fortified bouillon on micronutrient status should either employ a cap on rations of multiple micronutrient-fortified bouillon or reformulate the bouillon to account for possible increases in consumption when bouillon is provided under the auspices of a clinical trial.

The strengths of the study were the combination of systematic center-based sensory evaluations among typical purchasers and consumers of bouillon with an in-home acceptability study, which allowed participants the opportunity to use and evaluate the bouillon in their daily cooking for 2 wk and gain experiences with the bouillon. Moreover, although we did not conduct formal qualitative data analysis, we found that the quantitative results generally were supported by comments from the focus group participants.

However, the study also has several limitations. First, we did not collect data on intrahousehold distribution of bouillon and are therefore unable to calculate individual intake. In addition, because bouillon use and consumption were not observed, estimates based on wrapper counts may overestimate actual bouillon intake (e.g., underreporting of sharing, no accounting for food waste, etc.). Second, bias in reporting is possible. Social desirability bias may have led to underreporting of sharing bouillon cubes outside of the household, as well as a possible reluctance among the study participants to give negative ratings (hedonic responses) as to the acceptability of the cubes [41]. The fact that ∼ 90% of study participants rated all 3 bouillon formulations as either “like” or “like very much,” combined with high product disappearance rates during the in-home portion of the study, lend weight to the assertion that both the control (fortified with iodine only) and multiple micronutrient-fortified bouillon cubes were well received and highly acceptable. However, more than 1/3 of index participants attributed positive health effects to the study-provided bouillon cubes, possibly because they were provided by a nutrition research team, which may have also biased reported acceptability. Third, the acceptability study was of short duration (2 wk); it is unknown if the acceptability and use of the study-provided bouillon cubes would change when the products are provided for a longer period. However, reports in the literature for similar products used in the context of research trials (e.g., double-fortified salt, multiple micronutrient powders, small-quantity lipid-based nutrient supplements) have indicated that acceptability and consumption can remain high over longer periods of time [[42], [43], [44]]. Finally, all 3 cubes included in this study were specifically developed for this study. Thus, although the cubes were formulated as a model nonproprietary bouillon cube by an international consortium of bouillon producers, no commercially available cubes were provided as a reference in this study.

These results may have important and timely implications for policy discussions around the multiple micronutrient fortification of bouillon cubes [7]. The production of multiple micronutrient-fortified bouillon cubes was possible, and the inclusion of multiple micronutrients at the levels evaluated in this study did not cause organoleptic issues in the context of this acceptability trial, both of which are important considerations in the design and implementation of bouillon fortification programs. However, the current study did not incorporate the storage duration and conditions to which commercial bouillon products are exposed, from production to consumption; this information is required to understand the feasibility of multiple micronutrient-fortified commercial bouillon products. This would require the long-term stability and organoleptic properties of multiple micronutrient-fortified bouillon cubes to be tested under stressful conditions (e.g., heat, humidity).

Further to technical feasibility, policy discussions for bouillon fortification also need to balance efficacy, safety, and economic factors. As many countries in West Africa are facing an increasing double burden of disease (i.e., high prevalence of micronutrient deficiencies as well as rising prevalences of obesity and non-communicable diseases), attention must be paid to the role of bouillon as a source of dietary salt and the perception of multiple micronutrient-fortified bouillon as a food which “improves health” and “encourages eating.” Our prior work in this region evaluated the contribution of bouillon to total salt intake and found that bouillon contributed < 25% of household daily salt intake, even without considering the contribution of processed foods to salt intake [45]. We are not aware of any evidence suggesting that mandatory fortification of a staple food or condiment increases its consumption, which, in the case of bouillon, is likely influenced by taste preferences as well as income. However, this is an important question for future research. Finally, the results of the hypothetical WTP analyses suggest that the experiences of the consumers who participated in this acceptability trial did not increase the perceived value of the micronutrients that could be included in fortified bouillon cubes. On the one hand, this is not unexpected [46], but it may pose commercial and policy concerns because large-scale food fortification programs generally pass along premix and other program costs to consumers; following that cost-pass-through pattern in the case of bouillon cubes might be challenging. However, this was a small study that focused primarily on acceptability. Further research is needed to understand how micronutrient fortification may affect WTP.

In conclusion, the findings of this acceptability study indicate that all 3 formulations of bouillon cubes assessed are acceptable to women and their households in 2 districts in northern Ghana. The high acceptability and reported consumption of the tested products and the high prevalence of micronutrient deficiencies among women of reproductive age and young children in Northern Region, Ghana, supports the need to investigate the efficacy of multiple micronutrient-fortified bouillon cubes to improve micronutrient status of these populations.

## Author contributions

The authors’ responsibilities were as follows—KRW, SJZ, CDA, SAV, KPA, MJH, SAA, and RES designed research; SMK, EB, JND, and KWN conducted research; KRW, CDA, and XT analyzed data and performed statistical analysis; KRW and RES wrote the paper and had primary responsibility for final content. All authors have read and agreed to the published version of the manuscript.

## Conflict of interest

Reina Engle-Stone is an Editorial Board Member of Current Developments in Nutrition and played no role in the Journal’s evaluation of the manuscript. All other authors declare no competing interests.

## Funding

This work was supported, in whole or in part, by a grant from Helen Keller International (66504-UCD-01) through support from the Bill & Melinda Gates Foundation (INV007916) to the University of California, Davis. Under the grant conditions of the Foundation, a Creative Commons Attribution 4.0 Generic License has already been assigned to the Author Accepted Manuscript version that might arise from this submission.

## Data availability

Data described in the manuscript, code book, and analytic code will be made publicly and freely available without restriction at https://osf.io/t3zrnk/.

## Footnotes

1 In this context, describing food as “tasty” means it falls within the range of the socially accepted norm for the way a particular food is expected to taste. If a food is said to be “sweet”, it is considered to exceed the upper limit of the expected norm (i.e., “super tasty”), and is considered unhealthy.
