# Supplementary material for: Acceptability of Multiple Micronutrient-Fortified Bouillon Cubes among Women and Their Households in 2 Districts in The Northern Region of Ghana
Source: Curr Dev Nutr. 2023 Dec 8;8(1):102056. doi: 10.1016/j.cdnut.2023.102056 (PMC10832376; doi:10.1016/j.cdnut.2023.102056)
Supplement: Multimedia component 1 [file mmc1.pptx]

## Slide 1
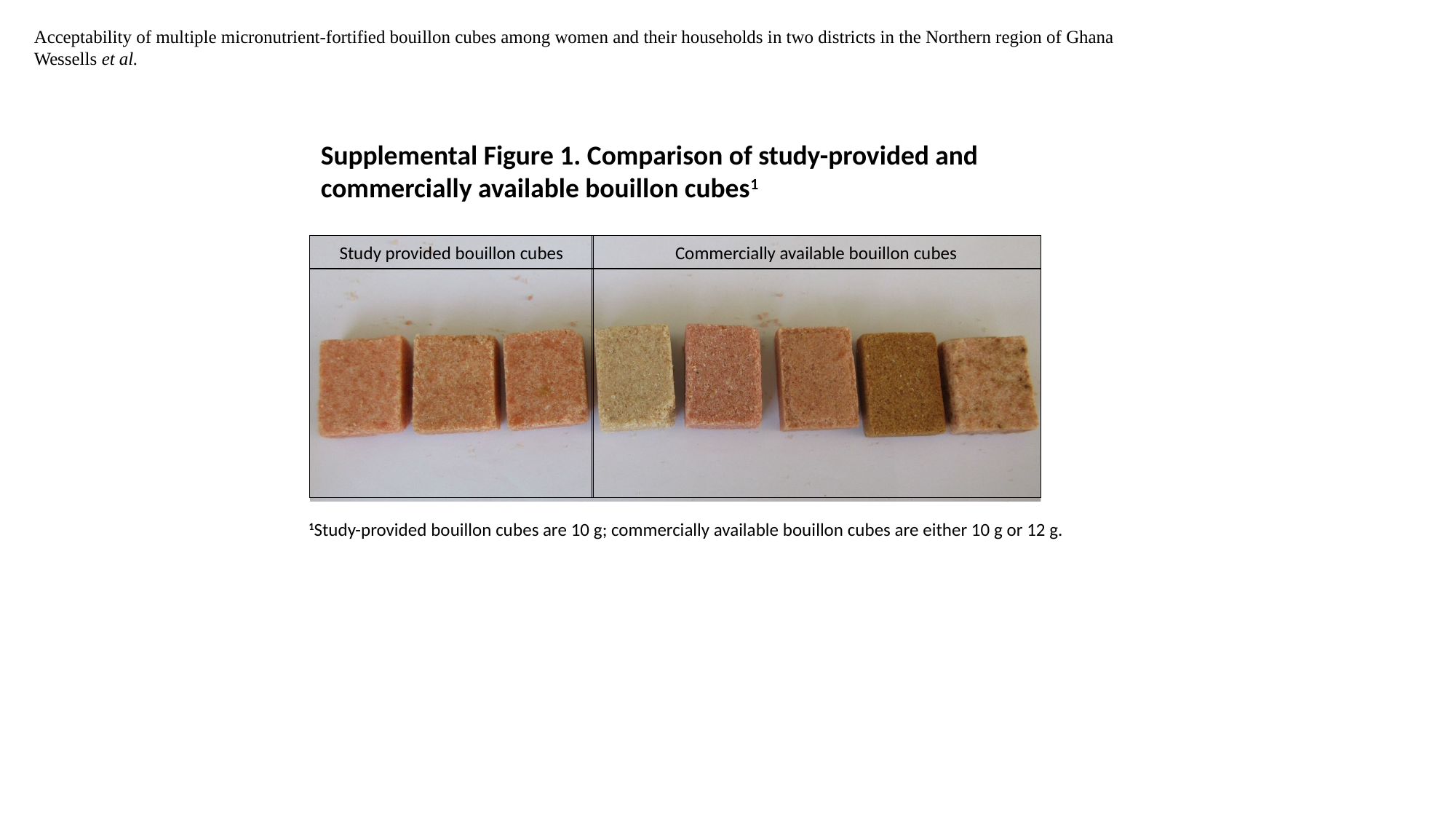

Acceptability of multiple micronutrient-fortified bouillon cubes among women and their households in two districts in the Northern region of Ghana
Wessells et al.
Supplemental Figure 1. Comparison of study-provided and commercially available bouillon cubes1
Commercially available bouillon cubes
Study provided bouillon cubes
1Study-provided bouillon cubes are 10 g; commercially available bouillon cubes are either 10 g or 12 g.
