# Supplementary material for: Acceptability of Multiple Micronutrient-Fortified Bouillon Cubes among Women and Their Households in 2 Districts in The Northern Region of Ghana
Source: Curr Dev Nutr. 2023 Dec 8;8(1):102056. doi: 10.1016/j.cdnut.2023.102056 (PMC10832376; doi:10.1016/j.cdnut.2023.102056)
Supplement: Multimedia component 2 [file mmc2.docx]

**Supplementary Material**

**Supplemental Methods.** Standardized recipes for dried okra soup and jollof rice.

**Supplemental Table 1.** Center-based evaluations of acceptability among different formulations of multiple micronutrient-fortified bouillon cubes: “Liked” vs. “Not-Liked” as a dichotomous outcome

**Supplemental Table 2.** Center-based evaluations of acceptability, using “just-about-right” questions, among different formulations of multiple micronutrient-fortified bouillon cubes

**Supplemental Table 3.** Center-based evaluations of acceptability, using a “check all that apply” questionnaire, among different formulations of multiple micronutrient-fortified bouillon cubes

**Supplemental Table 4.** In-home evaluations at endline of acceptability among different formulations of multiple micronutrient-fortified bouillon cubes: “Liked” vs. “Not-Liked” as a dichotomous outcome

**Supplemental Table 5.**  In-home evaluations at endline of acceptability, using “just-about-right” questions, among different formulations of multiple micronutrient-fortified bouillon cubes

**Supplemental Table 6**. In-home evaluations of acceptability at endline, using a “check all that apply” questionnaire, among different formulations of multiple micronutrient-fortified bouillon cubes

**Supplemental Table 7.** Micronutrient concentrations of “upper-level” multiple micronutrient-fortified bouillon cubes post-acceptability trial

**Supplemental Methods. Standardized recipes for dried okra soup and jollof rice.**

**Standardized dried okra soup recipe (ingredients listed in order of addition to the recipe)**

| **Ingredient** | **Weight in grams** | **Cooking methods** |
| --- | --- | --- |
| Groundnut paste^#^ | 200.0 | Cooked down until oil from groundnut floats to the top |
| Water | 1800.0 | Boiling |
| Dawadawa | 50.0 | Boiling |
| Fresh tomato pepper mix* | 200.0 | Boiling |
| Powdered anchovies | 120.0 | Boiling |
| Bouillon cubes | 20.0 | Boiling |
| Salt | 30.0 | Boiling |
| Water | 2000.0 | Boiling |
| Powdered dried okro | 60.0 | Boil until desired thickness |

^#^The groundnut paste was mixed with about 200 g of water to form a slurry. The 200 g of water is part of the total volume of water used to make the dish, which is 4000 g.

**Standardized jollof rice recipe (ingredients listed in order of addition to the recipe)**

| **Ingredient** | **Weight in grams** | **Cooking methods** |
| --- | --- | --- |
| Cooking oil | 250.0 |  |
| Diced onions | 50.0 | Frying until translucent |
| Tomato paste | 360.0 | Frying. Cooked until the added oil floats back on top of the mix |
| Fresh tomato pepper mix***** | 420.0 | Frying. Cooked until the added oil floats back on top of the mix |
| Powdered anchovies | 150.0 | Added to sauce |
| Bouillon cubes | 20.0 | Added to sauce |
| Salt | 30.0 | Added to sauce |
| Water | 4000.0 | Rolling boil |
| Raw rice | 1500.0 | Boil until grains absorb about 90% of water. Steam until rice is fluffy. |

# Fresh tomato pepper mix

| **Ingredient** | **Weight in grams** |
| --- | --- |
| Fresh pepper without waste | 65.0 |
| Onions without waste | 170.0 |
| Fresh tomato without waste | 1220.0 |
| Water | 80.0 |

**Supplemental Table 1. Center-based evaluations of acceptability among different formulations of multiple micronutrient-fortified bouillon cubes: “Liked” vs. “Not-Liked” as a dichotomous outcome**

|  | **Bouillon cube formulation^1^** | | |  |
| --- | --- | --- | --- | --- |
| **Variable** | **“Upper-level”** | **“Lower-level”** | **Control** | **P-value^2^** |
| Participants, n | 83 | 83 | 83 |  |
| **Acceptability** |  |  |  |  |
| **Dry bouillon, n (%) “liked” ^3^** |  |  |  |  |
| Overall liking | 74 (89.2) | 80 (96.4) | 79 (95.2) | 0.16 |
| Appearance | 76 (91.6) | 75 (90.4) | 79 (95.2) | 0.31 |
| Feel | 76 (91.6) | 79 (95.2) | 78 (94.0) | 0.62 |
| Crumble | 71 (85.5) ^ab^ | 78 (94.0) ^a^ | 81 (97.6) ^b^ | 0.01 |
| Aroma | 73 (88.0) | 74 (89.2) | 76 (91.6) | 0.69 |
| Taste | 77 (92.8) | 78 (94.0) | 80 (96.4) | 0.52 |
| **Prepared bouillon (dried okra soup), n (%) “liked”** |  |  |  |  |
| Overall liking | 74 (89.2) ^ab^ | 78 (94.0) ^a^ | 82 (98.8) ^b^ | 0.01 |
| Appearance | 79 (95.2) | 77 (92.8) | 82 (98.8) | 0.06 |
| Color | 80 (96.4) | 77 (92.8) | 81 (97.6) | 0.25 |
| Aroma | 76 (91.6) | 77 (92.8) | 79 (95.2) | 0.47 |
| Taste | 76 (91.6) | 74 (89.2) | 78 (94.0) | 0.34 |
| Saltiness | 75 (90.4) | 76 (91.6) | 76 (91.6) | 0.70 |
| Aftertaste | 67 (80.7) | 71 (85.5) | 73 (88.0) | 0.27 |
| **Prepared bouillon (jollof rice), n (%) “liked”** |  |  |  |  |
| Overall liking | 77 (92.8) | 79 (95.2) | 76 (91.6) | 0.67 |
| Appearance | 79 (95.2) | 79 (95.2) | 79 (95.2) | 0.99 |
| Color | 80 (96.4) | 79 (95.2) | 80 (96.4) | 0.90 |
| Aroma | 80 (96.4) | 81 (97.6) | 76 (91.6) | 0.15 |
| Taste | 81 (97.6) | 77 (92.8) | 79 (95.2) | 0.38 |
| Saltiness | 78 (94.0) | 75 (90.4) | 74 (89.2) | 0.49 |
| Aftertaste | 67 (80.7) | 64 (77.1) | 68 (81.9) | 0.68 |

^1^ Values n (%). “Upper-level” multiple micronutrient fortified bouillon cubes were fortified with vitamin A (200 µg RE/g), folic acid (80 µg/g), vitamin B12 (1.2 µg/g), iron (4 mg/g), zinc (3 mg/g), and iodine (30 µg/g). “Lower-level” multiple micronutrient fortified bouillon cubes were fortified with vitamin A (96 µg RE/g), folic acid (28.8 µg/g), vitamin B12 (0.288 µg/g), iron (1.3 mg/g), zinc (1.68 mg/g), and iodine (30 µg/g). The control bouillon cube was fortified with iodine (30 µg/g) only.

^2^P-values from modified Poisson regression, followed by post-hoc pairwise comparisons to estimate prevalence ratios, indicated by superscripts if the outcome was found to differ.

^3^Overall liking and acceptability of various organoleptic characteristics were assessed using a 5-point Likert scale: 1 = Dislike very much, 2 = Dislike, 3 = Neither like nor dislike, 4 = Like, 5 = Like very much. In this analysis, participants’ responses were re-classified into categories of “liked” (scores of 4 or 5) and “not-liked” (scores of 1-3).

**Supplemental Table 2. Center-based evaluations of acceptability, using “just-about-right” questions, among different formulations of multiple micronutrient-fortified bouillon cubes**

|  | **Bouillon cube formulation^1^** | | |  |
| --- | --- | --- | --- | --- |
| **Variable** | **“Upper-level”** | **“Lower-level”** | **Control** | **P-value^2^** |
| Participants, n | 83 | 83 | 83 |  |
| **Dry bouillon, n (%) “just-about-right”^3^** |  |  |  |  |
| Color | 63 (75.9) | 63 (75.9) | 71 (85.5) | 0.18 |
| Texture (smoothness, fineness of particles) | 52 (62.7) ^a^ | 56 (67.5) ^ab^ | 66 (79.5) ^b^ | 0.02 |
| Hardness | 56 (67.5) | 60 (72.3) | 61 (73.5) | 0.53 |
| Stickiness | 51 (61.4) | 52 (62.7) | 50 (60.2) | 0.94 |
| Saltiness | 68 (81.9) | 70 (84.3) | 74 (89.2) | 0.26 |
| **Prepared bouillon (dried okra soup) , n (%) “just-about-right”** |  |  |  |  |
| Saltiness | 74 (89.2) | 72 (86.7) | 67 (80.7) | 0.42 |
| Aftertaste | 58 (69.9) | 59 (71.1) | 52 (62.7) | 0.42 |
| **Prepared bouillon (jollof rice) , n (%) “just-about-right”** |  |  |  |  |
| Saltiness | 71 (85.5) | 73 (88.0) | 66 (79.5) | 0.27 |
| Aftertaste | 53(63.9) ^b^ | 42 (50.6 )^a^ | 52 (62.7) ^b^ | 0.04 |

^1^ Values n (%). “Upper-level” multiple micronutrient fortified bouillon cubes were fortified with vitamin A (200 µg RE/g), folic acid (80 µg/g), vitamin B12 (1.2 µg/g), iron (4 mg/g), zinc (3 mg/g), and iodine (30 µg/g). “Lower-level” multiple micronutrient fortified bouillon cubes were fortified with vitamin A (96 µg RE/g), folic acid (28.8 µg/g), vitamin B12 (0.288 µg/g), iron (1.3 mg/g), zinc (1.68 mg/g), and iodine (30 µg/g). The control bouillon cube was fortified with iodine (30 µg/g) only.

^2^P-values from modified Poisson regression, followed by post-hoc pairwise comparisons to estimate prevalence ratios, indicated by superscripts if the outcome was found to differ.

^3^Just-about-right outcomes were analyzed as dichotomous variables by re-classifying participants into either ‘right’ (score of just-about-right) vs ‘not right’ (score of either too much or too little).

**Supplemental Table 3. Center-based evaluations of acceptability, using a “check all that apply” questionnaire, among different formulations of multiple micronutrient-fortified bouillon cubes**

|  | **Multiple micronutrient-fortified bouillon cube formulation^1^** | | |  |
| --- | --- | --- | --- | --- |
| **Variable** | **“Upper-level”** | **“Lower-level”** | **Control** | **P-value^2^** |
| Participants, n | 83 | 83 | 83 |  |
| **Dry bouillon** |  |  |  |  |
| Salty | 66 (79.5) | 64 (77.1) | 65 (78.3) | 0.24 |
| Metallic taste | 4 (4.8) | 3 (3.6) | 3 (3.6) | 0.87 |
| Rancid taste | 1 (1.2) | 0 (0.0) | 1 (1.2) | --- |
| Bitter | 0 (0.0) | 0 (0.0) | 0 (0.0) | --- |
| Bland | 0 (0.0) | 1 (1.2) | 1 (1.2) | --- |
| Too much shrimp-flavor | 27 (32.5) | 15 (18.1) | 20 (24.1) | 0.05 |
| Off-flavor | 4 (4.8) | 7 (8.4) | 5 (6.0) | 0.30 |
| Fatty | 8 (9.6) | 7 (8.4) | 6 (7.2) | 0.83 |
| Aftertaste | 47 (56.6) | 45 (54.2) | 46 (55.4) | 0.92 |
| Umami | 72 (86.7) | 76 (91.6) | 72 (86.7) | 0.33 |
| Too much bouillon flavor | 18 (21.7) | 18 (21.7) | 19 (22.9) | 0.95 |
| Too little bouillon flavor | 8 (9.6) | 8 (9.6) | 7 (8.4) | 0.96 |
| **Prepared bouillon (dried okra soup)** |  |  |  |  |
| Salty | 62 (74.7) | 63 (75.9) | 63 (75.9) | 0.88 |
| Metallic taste | 2 (2.4) | 0 (0.0) | 4 (4.8) | --- |
| Rancid taste | 0 (0.0) | 0 (0.0) | 0 (0.0) | --- |
| Bitter | 0 (0.0) | 2 (2.4) | 0 (0.0) | --- |
| Bland | 0 (0.0) | 4 (4.8) | 1 (1.2) | --- |
| Too much shrimp-flavor | 19 (22.9) ^b^ | 11 (13.3) ^a^ | 16 (19.3) ^ab^ | 0.03 |
| Off-flavor | 6 (7.2) | 2 (2.4) | 5 (6.0) | 0.31 |
| Fatty | 3 (3.6) | 5 (6.0) | 5 (6.0) | 0.59 |
| Aftertaste | 53 (63.9) | 56 (67.5) | 53 (63.9) | 0.60 |
| Umami | 73 (88.0) | 74 (89.2) | 70 (84.3) | 0.30 |
| Too much bouillon flavor | 12 (14.5) | 7 (8.4) | 6 (7.2) | 0.30 |
| Too little bouillon flavor | 13 (15.7) | 15 (18.1) | 16 (19.3) | 0.80 |
| **Prepared bouillon (jollof rice)** |  |  |  |  |
| Salty | 62 (74.7) | 60 (72.3) | 61 (73.5) | 0.22 |
| Metallic taste | 4 (4.8) | 3 (3.6) | 2 (2.4) | 0.30 |
| Rancid taste | 0 (0.0) | 1 (1.2) | 0 (0.0) | --- |
| Bitter | 0 (0.0) | 0 (0.0) | 1 (1.2) | --- |
| Bland | 2 (2.4) | 2 (2.4) | 2 (2.4) | 1.00 |
| Too much shrimp-flavor | 19 (22.9) | 19 (22.9) | 17 (20.5) | 0.81 |
| Off-flavor | 3 (3.6) | 1 (1.2) | 4 (4.8) | 0.28 |
| Fatty | 10 (12.0) | 9 (10.8) | 6 (7.2) | 0.55 |
| Aftertaste | 47 (56.6) | 46 (55.4) | 44 (53.0) | 0.75 |
| Umami | 69 (83.1) | 69 (83.1) | 71 (85.5) | 0.76 |
| Too much bouillon flavor | 8 (9.6) | 11 (13.3) | 7 (8.4) | 0.43 |
| Too little bouillon flavor | 12 (14.5) | 10 (12.0) | 13 (15.7) | 0.64 |

^1^ Values n (%). “Upper-level” multiple micronutrient fortified bouillon cubes were fortified with vitamin A (200 µg RE/g), folic acid (80 µg/g), vitamin B12 (1.2 µg/g), iron (4 mg/g), zinc (3 mg/g), and iodine (30 µg/g). “Lower-level” multiple micronutrient fortified bouillon cubes were fortified with vitamin A (96 µg RE/g), folic acid (28.8 µg/g), vitamin B12 (0.288 µg/g), iron (1.3 mg/g), zinc (1.68 mg/g), and iodine (30 µg/g). The control bouillon cube was fortified with iodine (30 µg/g) only.

^2^P-values from modified Poisson regression, followed by post-hoc pairwise comparisons to estimate prevalence ratios, indicated by superscripts if the outcome was found to differ.

**Supplemental Table 4. In-home evaluations at endline of acceptability among different formulations of multiple micronutrient-fortified bouillon cubes: “Liked” vs. “Not-Liked” as a dichotomous outcome**

|  | **Bouillon cube formulation^1^** | | |  |
| --- | --- | --- | --- | --- |
| **Variable** | **“Upper-level”** | **“Lower-level”** | **Control** | **P-value^2^** |
| Participants^3^, n | 26 | 27 | 27 |  |
| **Overall acceptability, n (%) “liked”^4^** |  |  |  |  |
| Index participant | 24 (96.0) | 24 (88.9) | 27 (100) | 0.91 |
| Household members | 22 (88.0) | 25 (92.6) | 27 (100) | 0.90 |
| **Dry bouillon, n (%) “liked”** |  |  |  |  |
| Appearance | 25 (100) | 27 (100) | 27 (100) | --- |
| Feel | 25 (100) | 27 (100) | 27 (100) | --- |
| Crumble | 25 (100) | 27 (100) | 27 (100) | --- |
| Aroma | 23 (92.0) | 24 (88.9) | 24 (88.9) | 0.99 |
| Taste | 25 (100) | 27 (100) | 27 (100) | --- |
| Packaging | 23 (92.0) | 27 (100) | 23 (85.2) | 0.85 |
| **Prepared bouillon (household dishes), n (%) “liked”** |  |  |  |  |
| Appearance | 22 (88.0) | 26 (96.3) | 26 (96.3) | 0.94 |
| Color | 22 (88.0) | 24 (88.9) | 24 (88.9) | 1.00 |
| Aroma | 24 (96.0) | 25 (92.6) | 26 (96.3) | 0.99 |
| Taste | 25 (100) | 27 (100) | 27 (100) | --- |
| Saltiness | 25 (100) | 27 (100) | 27 (100) | --- |
| Aftertaste | 24 (96.0) | 26 (96.3) | 24 (88.9) | 0.95 |

^1^ Values n (%). “Upper-level” multiple micronutrient fortified bouillon cubes were fortified with vitamin A (200 µg RE/g), folic acid (80 µg/g), vitamin B12 (1.2 µg/g), iron (4 mg/g), zinc (3 mg/g), and iodine (30 µg/g). “Lower-level” multiple micronutrient fortified bouillon cubes were fortified with vitamin A (96 µg RE/g), folic acid (28.8 µg/g), vitamin B12 (0.288 µg/g), iron (1.3 mg/g), zinc (1.68 mg/g), and iodine (30 µg/g). The control bouillon cube was fortified with iodine (30 µg/g) only.

^2^P-values from modified Poisson regression, followed by post-hoc pairwise comparisons to estimate prevalence ratios, indicated by superscripts if the outcome was found to differ.

^3^Data unavailable for one participant in the “lower-level” bouillon cube formulation group.

^4^Overall liking and acceptability of various organoleptic characteristics were assessed using a 5-point Likert scale: 1 = Dislike very much, 2 = Dislike, 3 = Neither like nor dislike, 4 = Like, 5 = Like very much. In this analysis, participants’ responses were re-classified into categories of “liked” (scores of 4 or 5) and “not-liked” (scores of 1-3).

**Supplemental Table 5. In-home evaluations at endline of acceptability, using “just-about-right” questions, among different formulations of multiple micronutrient-fortified bouillon cubes**

|  | **Bouillon cube formulation^1^** | | |  |
| --- | --- | --- | --- | --- |
| **Variable** | **“Upper level”** | **“Lower level”** | **Control** | **P-value^2^** |
| Participants^3^, n | 26 | 27 | 27 |  |
| **Dry bouillon, n (%) “just-about-right”^4^** |  |  |  |  |
| Color | 23 (92.0) | 26 (96.3) | 26 (96.3) | 0.98 |
| Texture (smoothness, fineness of particles) | 24 (96.0) | 26 (96.3) | 25 (92.6) | 0.99 |
| Hardness | 23 (92.0) | 24 (88.9) | 19 (70.4) | 0.65 |
| Stickiness | 22 (88.0) | 22 (81.5) | 19 (70.4) | 0.77 |
| Saltiness | 19 (76.0) | 20 (74.1) | 20 (74.1) | 1.00 |
| **Prepared bouillon (household dishes), n (%) “just-about-right”** |  |  |  |  |
| Saltiness | 20 (80.0) | 18 (66.7) | 18 (66.7) | 0.81 |
| Aftertaste | 19 (76.0) | 17 (63.0) | 18 (66.7) | 0.84 |

^1^ Values n (%). “Upper-level” multiple micronutrient fortified bouillon cubes were fortified with vitamin A (200 µg RE/g), folic acid (80 µg/g), vitamin B12 (1.2 µg/g), iron (4 mg/g), zinc (3 mg/g), and iodine (30 µg/g). “Lower-level” multiple micronutrient fortified bouillon cubes were fortified with vitamin A (96 µg RE/g), folic acid (28.8 µg/g), vitamin B12 (0.288 µg/g), iron (1.3 mg/g), zinc (1.68 mg/g), and iodine (30 µg/g). The control bouillon cube was fortified with iodine (30 µg/g) only.^2^P-values from modified Poisson regression, followed by post-hoc pairwise comparisons to estimate prevalence ratios, indicated by superscripts if the outcome was found to differ.

^3^Data unavailable for one participant in the “lower-level” bouillon cube formulation group.

^4^Just-about-right outcomes were analyzed as dichotomous variables by re-classifying participants into either ‘right’ (score of just-about-right) vs ‘not right’ (score of either too much or too little).

**Supplemental Table 6. In-home evaluations of acceptability at endline, using a “check all that apply” questionnaire, among different formulations of multiple micronutrient-fortified bouillon cubes**

|  | **Bouillon cube formulation^1^** | | |  |
| --- | --- | --- | --- | --- |
| **Variable** | **“Upper-level”** | **“Lower-level”** | **Control** | **P-value^2^** |
| Participants^3^, n | 26 | 27 | 27 |  |
| **Dry bouillon, n (%)** |  |  |  |  |
| Salty | 10 (40.0) | 11 (40.7) | 13 (48.1) | 0.88 |
| Metallic taste | 0 (0.0) | 0 (0.0) | 0 (0.0) | --- |
| Rancid taste | 0 (0.0) | 0 (0.0) | 0 (0.0) | --- |
| Bitter | 0 (0.0) | 0 (0.0) | 0 (0.0) | --- |
| Bland | 0 (0.0) | 0 (0.0) | 0 (0.0) | --- |
| Too much shrimp-flavor | 1 (4.0) | 2 (7.4) | 2 (7.4) | 0.86 |
| Off-flavor | 0 (0.0) | 0 (0.0) | 0 (0.0) | --- |
| Fatty | 0 (0.0) | 4 (14.8) | 0 (0.0) | --- |
| Aftertaste | 10 (40.0) | 11 (40.7) | 13 (48.1) | 0.88 |
| Umami | 17 (62.0) | 22 (81.5) | 23 (85.2) | 0.77 |
| Too much bouillon flavor | 3 (12.0) | 4 (14.8) | 3 (11.1) | 0.92 |
| Too little bouillon flavor | 0 (0.0) | 2 (7.4) | 0 (0.0) | --- |
| **Prepared bouillon (household recipes), n (%)** |  |  |  |  |
| Salty | 10 (40.0) | 11 (40.7) | 13 (48.1) | 0.88 |
| Metallic taste | 0 (0.0) | 0 (0.0) | 0 (0.0) |  |
| Rancid taste | 0 (0.0) | 0 (0.0) | 0 (0.0) |  |
| Bitter | 0 (0.0) | 0 (0.0) | 0 (0.0) |  |
| Bland | 0 (0.0) | 0 (0.0) | 0 (0.0) |  |
| Too much shrimp-flavor | 0 (0.0) | 2 (7.4) | 0 (0.0) |  |
| Off-flavor | 0 (0.0) | 0 (0.0) | 0 (0.0) |  |
| Fatty | 0 (0.0) | 1 (3.7) | 2 (7.4) |  |
| Aftertaste | 10 (40.0) | 10 (37.0) | 13 (48.1) | 0.81 |
| Umami | 14 (56.0) | 18 (66.7) | 20 (74.1) | 0.73 |
| Too much bouillon flavor | 2 (8.0) | 3 (11.1) | 1 (3.7) | 0.64 |
| Too little bouillon flavor | 4 (16.0) | 3 (11.1) | 2 (7.4) | 0.67 |

^1^ Values n(%). “Upper-level” multiple micronutrient fortified bouillon cubes were fortified with vitamin A (200 µg RE/g), folic acid (80 µg/g), vitamin B12 (1.2 µg/g), iron (4 mg/g), zinc (3 mg/g), and iodine (30 µg/g). “Lower-level” multiple micronutrient fortified bouillon cubes were fortified with vitamin A (96 µg RE/g), folic acid (28.8 µg/g), vitamin B12 (0.288 µg/g), iron (1.3 mg/g), zinc (1.68 mg/g), and iodine (30 µg/g). The control bouillon cube was fortified with iodine (30 µg/g) only.

^2^P-values from modified Poisson regression, followed by post-hoc pairwise comparisons to estimate prevalence ratios, indicated by superscripts if the outcome was found to differ.

^3^Data unavailable for one participant in the “lower-level” bouillon cube formulation group.

**Supplemental Table 7. Micronutrient concentrations of “upper-level” multiple micronutrient-fortified bouillon cubes post-acceptability trial^1^**

| **Micronutrient** | **Target^2^** | **Controlled conditions** | **Controlled conditions, 1 h boiling** | **Ambient conditions** | **Ambient conditions, 1 h boiling** |
| --- | --- | --- | --- | --- | --- |
| Vitamin A (µg/g) | 200 | 210 + 2 | --- | 216 + 6 | --- |
| Vitamin B9 (µg/g) | 80 | 103.5 + 0.5 | 96.5 + 3.5 | 109.5 + 1.5 | 103.0 + 3.0 |
| Vitamin B12 (µg/g) | 1.2 | 1.19 + 0.1 | 1.17 + 0.01 | 1.26 + 0.06 | 1.20 + 0.01 |
| Iodine (µg/g) | 30 | 34 + 1 | 34.5 + 0.5 | --- | --- |

^1^Values mean + SD. After the completion of the acceptability trial (~11 months postproduction), micronutrient concentrations (vitamins A, B9, B12 and iodine) of the “upper-level” multiple micronutrient-fortified bouillon cubes (including both those stored under controlled conditions and those stored in ambient conditions in participants’ homes for two weeks) were measured by Eurofins Vitamin Testing Denmark A/S (Vejen, Denmark).

^2^Target micronutrient concentrations do not include overage values to account for micronutrient loss during storage and cooking. Overage values were selected based on industry experience and storage and cooking trials conducted by CSIRO and RISE: 30% for vitamin A, folic acid and vitamin B12, 0% for iron and zinc and 20% for iodine.
